# Experimental Study on Thermal Runaway Process of 18650 Lithium-Ion Battery under Different Discharge Currents

**DOI:** 10.3390/ma14164740

**Published:** 2021-08-22

**Authors:** Lun Li, Xiaoyu Ju, Xiaodong Zhou, Yang Peng, Zhizuan Zhou, Bei Cao, Lizhong Yang

**Affiliations:** 1 State Key Laboratory of Fire Science, University of Science and Technology of China, Hefei 230026, China; lilunust@mail.ustc.edu.cn (L.L.); zxd@ustc.edu.cn (X.Z.); py230@mail.ustc.edu.cn (Y.P.); zhizuan@mail.ustc.edu.cn (Z.Z.); caobei@ustc.edu.cn (B.C.); 2 Department of Mechanical Engineering, Toyohashi University of Technology, 1-1 Hibarigaoka, Tempaku, Toyohashi 441-8580, Japan

**Keywords:** lithium-ion batteries, thermal runaway, discharge condition, operational state, heat generation

## Abstract

Lithium-ion batteries (LIBs) subjected to external heat may be prone to failure and cause catastrophic safety issues. In this work, experiments were conducted to investigate the influence of discharge current on the thermal runaway process under thermal abuse. The calibrated external heat source (20 W) and discharge currents from 1 to 6 A were employed to match the thermal abuse conditions in an operational state. The results indicated that the key parameters during the failure process, such as the total mass loss, the onset temperatures of safety venting and thermal runaway, and the peak temperature, are ultimately determined by the capacity inside the battery, and the discharge current can hardly change it. However, discharge currents can produce extra energy to accelerate the thermal runaway process. Compared with the battery in an open circuit, the onset time of thermal runaway was reduced by 7.4% at 6 A discharge. To quantify the effect of discharge current, the total heat generation by discharge current was calculated. The results show that a heat generation of 1.6 kJ was produced when the battery was discharged at 6 A, which could heat the cell to 34 °C (neglect of heat loss). This study simulates the failure process of the LIB in the operational state, which is expected to help the safety application of LIB and improve the reliability of the battery management system.

## 1. Introduction

Lithium-ion batteries (LIBs) have become a promising choice for various electrical equipment, due to their high energy density, minimal memory effect, excellent cycle life, and continually reducing cost [1,2]. However, LIBs are mainly made of flammable electrolyte and active materials, which are active to react with each other exothermically when exposed to an abuse condition. Significant safety issues, which are related to fire and explosion hazards created from the LIBs’ failure, remain major obstacles for the large-scale application of LIBs. Battery faults, such as overheating, mechanical crash, overcharge, or short circuit [3,4], could deteriorate the safety performance of the battery and lead to catastrophic fire or explosion accidents. Therefore, it is essential to understand LIB failure mechanisms and assess their safety performance.

Thermal runaway is one of the most catastrophic battery failure phenomena, which refers to the uncontrollable exothermic chain reaction cause by a battery self-heating and is often accompanied by smoke generation, jet fire, or explosion [5]. So far, considerable research efforts have been dedicated to the thermal runaway mechanism of the battery. The thermal property of the battery components in a high-temperature environment has been investigated by thermal analysis tools such as accelerating rate calorimetry (ARC) [6,7], C80 calorimetry [8], vent size packet 2 (VSP2) adiabatic calorimetry [9,10,11], and differential scanning calorimetry (DSC) [12], and the internal reaction during the thermal runaway process was identified. As the temperature builds up, the battery undergoes the following reactions: the solid electrolyte interphase (SEI) layer decomposition, reaction between the anode material and electrolyte, reaction between the cathode material and electrolyte, electrolyte decomposition, and the reaction between the anode and the binder [5]. In addition, various combustion tests [13] have been conducted to explore the fire hazards and damage power of LIBs. Wang et al. [14] performed full-scale tests to investigate the combustion behavior of the LIB during the failure process. Mao et al. [15] investigated the combustion behavior of 18650-type LIB, and the influence mechanisms of state of charge (SOC) on LIB fire risk were revealed. All the aforementioned studies were conducted with the electrically isolated LIBs, while the thermal behaviors of the batteries in the operational state were rarely investigated.

Recently, battery safety incidents have been reported frequently during usage, i.e., in the operational state. The typical LIB fire accidents in the operational state are shown in Table 1. When current flows through a battery, the heat accumulates and the temperature of the battery rises steadily. The internal heat generations of the battery in the operational state have been extensively investigated in previous studies [16,17,18,19,20]. Kim [21] employed experimental and numerical methods to analyze the discharge behavior of LIB at different discharge rates. Their results indicated that the maximum temperature of the LIB during discharge was proportional to the current. Onda et al. [22,23] investigated the heat generation of a LIB during charging and discharging cycles and calculated the temperature rise. Their results showed that the cell temperature can reach up to 100 °C for a 3 C discharge rate. The previous research mainly focused on the heat generation of the battery in the operational state. However, the impact of heat generation on the thermal runaway process of the LIB was rarely studied.

Significant exothermic reactions during the discharge process made a difference between the electrically isolated LIB and the operational cell. The thermal runaway process of the LIB under discharge condition should be investigated to provide a comprehensive understanding of the operational cell. Furthermore, Wang et al. [28,29,30] conducted several experiments to analyze the discharge behavior of 18650 LIBs with external heat source and found that the probability of LIB thermal runaway occurrence decreased with the discharging current. However, this conclusion was ascribed to the consumed electricity during the discharge process, while the effect of discharge currents on the thermal runaway process is still unclear, which makes it difficult to evaluate battery safety in different discharge states.

Considering the aforementioned knowledge gap and the great significance of the safety design for LIBs in engineering applications, detailed studies for the thermal features of the battery in discharge conditions are essential. In this study, the thermal behavior of 18650 LIBs under different discharge conditions was investigated systematically. Different discharge currents were set to simulate the operational conditions of the LIB. The surface temperature, voltage variation, mass loss, as well as experimental phenomena were recorded during the test. This work aims to provide a comprehensive understanding of the thermal hazards of LIBs in the operational state and benefit the battery safety design.

## 2. Materials and Methods

### 2.1. Battery Samples

Commercial 18650-type cylindrical batteries (CGR18650CG, produced by Panasonic in Osaka, Japan) with the Li(Ni_0.33_Co_0.33_Mn_0.33_)O_2_ cathode and the graphite anode were tested in this study. The electrolyte solvent is mainly composed of alkyl carbonate. The samples are 18 mm in diameter and 65 mm in length. The nominal capacity and nominal voltage of these batteries are 2250 mAh and 3.6 V, respectively. Prior to each experiment, the plastic packaging of the cell was stripped off. The mass of a single cell without packaging was found to be 42.77 ± 0.05 g.

A battery cycler (CT-6 V 10mA, produced by Neware in Shenzhen, China) was used to charge/discharge the cells to the desired capacity. Before the test, the batteries were pre-cycled three times between 2.5 and 4.2 V at a current rate of 1 C (C-rate is an expression of current to normalize against battery capacity. For this sample, 1 C = 2.25 A). The cell was fully discharged and then charged to the desired SOCs. Subsequently, the batteries were placed for 24 h before the test to ensure better electrochemical stability.

### 2.2. Apparatus

**Figure 1:**
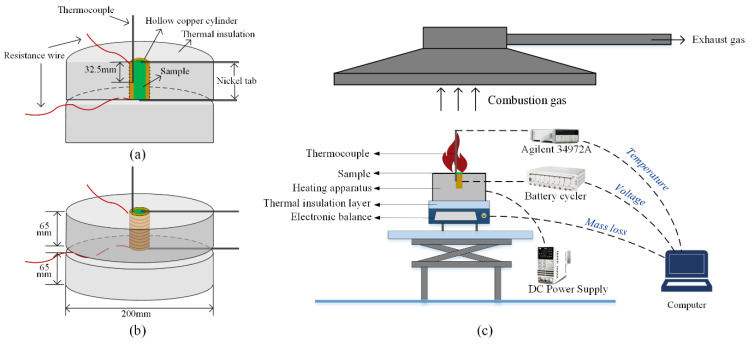
Vertical section view of the heating apparatus (**a**), Perspective view of the heating apparatus (**b**), and Schematic of the experimental setup (**c**).

The heating apparatus used to trigger LIBs thermal runaway is shown in Figure 1a,b, which was composed of a hollow copper cylinder, a resistive heating wire, and a ceramic fiber blanket [31]. The hollow cylinder, made of pure (99.5%) copper, was machined to snugly fit the sample studied in this work. The inner diameter, the outer diameter, and the height of the hollow cylinder are 18.04 mm, 22.02 mm, and 65.2 mm, respectively. Moreover, a K-type thermocouple with a diameter of 1 mm was pressed to the inner center of the hollow cylinder with a depth of 32.5 mm. The mounted thermocouple could be used to represent the surface temperature variation of the sample since a tight contact between the hollow cylinder and inserted battery. The hollow cylinder was tightly wrapped with a resistive heating wire (NI80-010-200, produced by OMEGA Engineering in Norwalk, CA, USA) after electrically insulated with 3M Isolant tape. A flame-retardant ceramic fiber blanket was employed to wrap the heated hollow cylinder to minimize the heat losses from the system to the environment. Only the top surface of the cell was open to the atmosphere to allow the escape of ejected battery materials. A similar apparatus was used to realize uniform heating and energetic quantification of a thermally induced LIB failure by other researchers [31,32].

The schematic of the experimental setup is shown in Figure 1c. A DC Power Supply (IT6721, produced by ITECH in Nanjing, China) was used to power the resistive wire with a constant value of 20 W. Temperature responses were recorded by Agilent 34972A with a frequency of 1 Hz. Moreover, the onset times of safety venting (*t_SV_*) and thermal runaway (*t_TR_)* were both recorded during the tests. The onset of safety venting is defined as the moment when there is a clear cracking sound and white aerosol ejecting from the safety vent ports, while the moment of the steep temperature rise is regarded as the onset of thermal runaway.

To implement a steady discharge and monitor the voltage history, both terminals of the tested sample were welded with a nickel tab and then connected to the battery cycler using high-temperature resistant wires. The mass of the cell during the test was measured by an electronic scale (XP10002S, produced by Mettler Toledo in Zurich, Switzerland) with an accuracy of 0.01 g. Moreover, the samples were weighed before and after each test to determine the accurate mass loss. Experimental processes were recorded by a digital camera (FDR-AX45, produced by Sony in Tokyo, Japan) at 25 fps.

### 2.3. Test Conditions

In this study, heating and discharging were started simultaneously, and the discharge process was stopped once the voltage of the battery dropped to zero. All experiments were performed at room temperature about 20 °C.

All the test conditions are shown in Table 2. The 75% SOC (1650 mAh) batteries were selected for the experiments. *C*_0_ represents the initial capacity before the test. The battery without discharge (i.e., in an open circuit) was tested in Group i, and the other two groups of tests were conducted with variations of discharge current (1 A, 2 A, 3 A, 4 A, and 6 A) and *C*_0_, respectively. The test were repeated at least three times for each condition to ensure reproducible results.

In Group ii, the samples with *C*_0_ = 1650 mAh were tested with different discharge currents. During the discharge process, the battery experienced an inevitable capacity decrease. The cell discharged at high rate lost more capacity compared to the one discharged at a low rate. Thus, the residual capacity inside the battery when the thermal runaway is triggered (*C_TR_*) varies with the discharge current in these tests. As mentioned in many previous studies, thermal runaway and heat effects in LIB cells are sensitive to their capacities (*C_TR_* in this paper) [33,34,35]. Therefore, the decrease of discharge currents and the capacity caused by discharge were both responsible for the thermal behavior of the samples in Group ii.

To eliminate the capacity difference brought by discharge current, different values of *C*_0_ were set to make *C_TR_* approximately equal to 1650 mA in Group iii. The value of *C*_0_ (as shown in Table 2) was selected based on our preliminary tests. The capacity variation and its uncertainty during the tests will be discussed in Section 3.3.

## 3. Results

### 3.1. Thermal Response of the LIB in an Open Circuit

**Figure 2:**
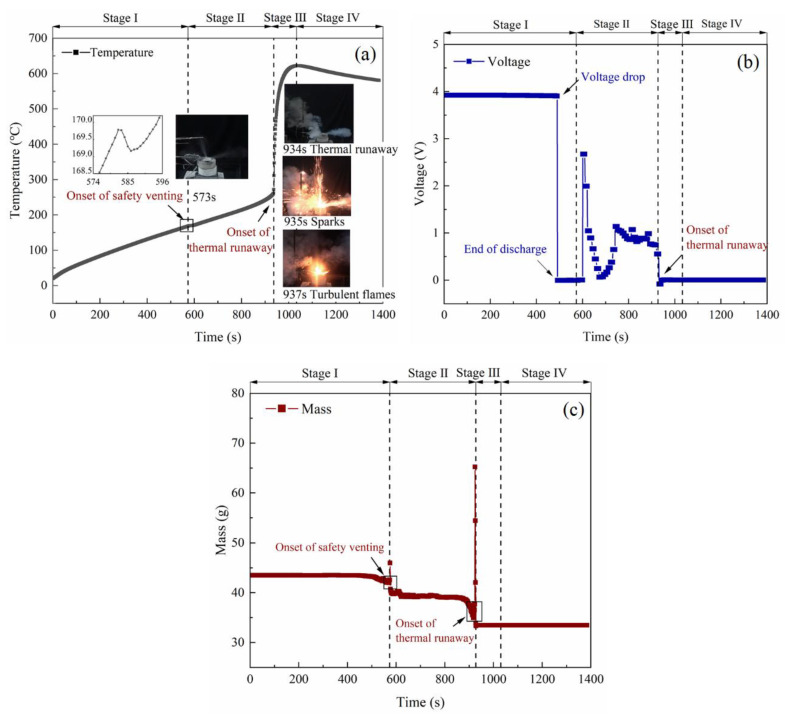
Test results from the battery during thermal runaway in an open circuit (Test 1). (**a**) The typical temperature curve and experimental phenomena. (**b**) The voltage characteristics across thermal runaway. (**c**) The representative mass history.

The temperature curve and experimental phenomena from the battery during thermal runaway in an open circuit are shown in Figure 2a. The thermal response of the LIB during the failure process can be roughly divided into four stages.

(1) Stage Ⅰ: The temperature of the cell increased linearly, and the heat mainly came from the external electric power. Once the cell temperature reached the onset temperature of the decomposition of the SEI layer, gases were gradually generated. After the cell temperature reached about 170 °C, the pressure inside the casing exceeded the threshold, leading to the open of the safety valve, and the excessive gases were released [36,37].

(2) Stage Ⅱ: A slight decrease in temperature was observed at 573 s, which was caused by the release of the hot gases [38] and the endothermic separator fusing [39]. Without the protection of the separator, intercalated lithium reacted directly with the organic electrolyte [40,41]. Then, the cell temperature started to increase quickly. The heat accumulation in this stage may be due to the SEI layer decomposition, reaction of intercalated lithium with electrolyte, cathode positive material decomposition [42], or the chemical cross over between anode and cathode [43].

(3) Stage Ⅲ: The cell underwent thermal runaway when the temperature reached about 262 °C. A considerable amount of combustible smoke was released violently. Some solid-phase particles were ejected in the form of sparks, igniting the combustible gases rapidly. Turbulent flame appeared at the safety vent ports, which lasted for approximately 1 min before it disappeared. Meanwhile, the cell temperature increased sharply to the maximum temperature.

(4) Stage Ⅳ: The exothermic reaction slows down, and the energy release is less than the heat loss. It was further observed that the cell temperature gradually dropped with the cooling effect of ambient environment.

The voltage characteristics during thermal runaways are shown in Figure 2b. Initially, the voltage showed a slight decrease (0.02 V) as the cell temperature increases, because the high temperature can speed up the degradation of cells [44]. Nevertheless, the voltage was maintained around 3.9 V and showed the integrity of the cell. When the temperature reached 153 °C, the voltage of the battery exhibited a sharp decline and stayed close to zero for about 93 s. This abrupt voltage drop can be attributed to the actions of the internal protection device inside the battery. As the pressure or the temperature inside the cell rose, an internal protection device was activated, which disconnected the external terminal from the cell, so that the voltage cannot be detected [45]. As the temperature increased, a recovery of voltage was observed after the internal protection device failed. During this time, the separator with ceramic coating loses its integrity in high temperature, and the battery cannot provide a stable voltage. Eventually, the voltage declined to zero permanently after the thermal runaway was triggered, and the internal structure of the battery had been destroyed completely. Similar voltage characteristics were also observed in previous studies [33,46,47].

The representative mass history of the battery is depicted in Figure 2c. The cell mass barely changed in stage Ⅰ, because the cell was well sealed before safety venting. After the safety vent ports were open, the cell mass continuously declines in high temperature. Eventually, the battery lost over 20% of its initial mass. These mass losses may result from the ejection of solid cathode and anode fragments, evaporation of the electrolyte, and internal chemical reaction. In addition, clear rebounds were observed at the onset of safety venting and thermal runaway, which was caused by the ejected gases exerting force on the electronic scale.

### 3.2. Discharge Tests on the LIBs with the Same C_0_

Samples with *C*_0_ = 1650 mAh were tested with different discharge currents in Group ii, and the capacity variations during the thermal runaway process are shown in Table 3. Almost 6–19% capacity was lost during the discharge process; therefore, the capacity lost can not be ignored in these tests. The different discharge currents and the capacity decrements were both responsible for the thermal behavior of the samples in Group ii.

**Figure 3:**
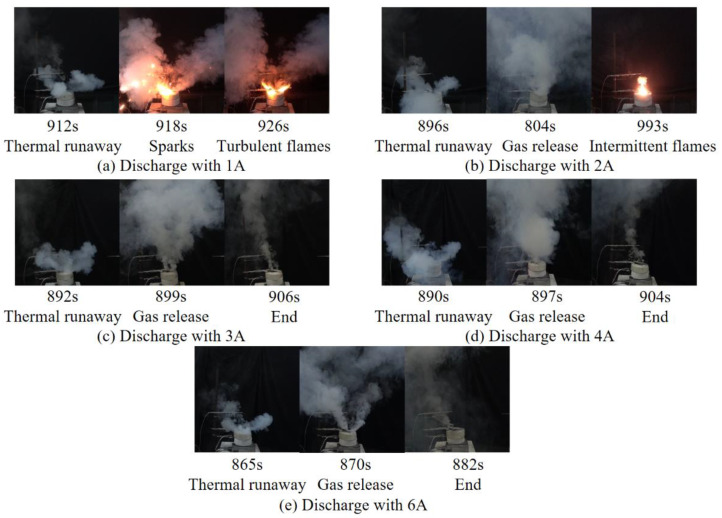
Thermal runaway behaviors under different discharge currents in Group ii. (**a**) The LIB discharge with 1 A; (**b**) The LIB discharge with 2 A; (**c**) The LIB discharge with 3 A; (**d**) The LIB discharge with 4 A; (**e**) The LIB discharge with 6 A.

Figure 3 presents the representative thermal runaway behaviors of the cells under different discharge currents in Group ii. For the batteries discharged at a relatively higher rate, the thermal runaway processes were less drastic. The cell went into thermal runaway without jet flame and considerable amounts of irritant smoke were observed only when discharged at 3 A, 4 A, and 6 A. The thermal runaway process of the LIBs discharging at low rate was more complex. When discharging at 2 A, intermittent flames were observed for a short time after gas emission. When the cell was discharged at 1 A, the combustible gases were ignited by sparks, and the turbulent flame was seen for approximately 10 s before its extinction. This phenomenon is similar to the thermal runaway behavior of the cell in an open circuit. It can be seen that a larger discharge current reduces the probability of the battery fire. However, the combustion reaction is complicated and highly dependent on the operating conditions.

**Figure 4:**
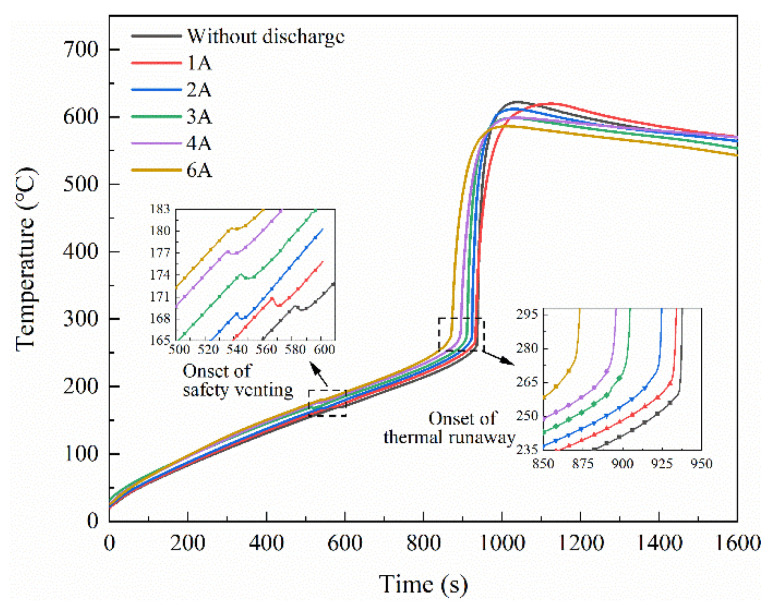
Typical temperature variation of the batteries under different discharge currents in Group ii.

The temperature variation of the batteries during the thermal runaway process in Group ii is shown in Figure 4; the test results in an open circuit (Test 1) were also included for comparison. The thermal responses of the LIBs under different discharge currents showed a similar tendency with the LIBs in an open circuit, which have been described in Section 3.1. Moreover, only a slight distinction was found on the temperature profile. Considering the inconsistency of the single battery and the experimental contingency, the key parameters of the thermal runaway process among three repeated tests are summarized in Table 4 to explore the effect of discharge current, where *T_SV_* and *T_TR_* represent the onset temperature of safety venting and thermal runaway, *T_max_* represents the maximum temperature achieved, (d*T*/d*t*)*_max_* represent the maximum temperature rise during the failure process, and the total mass loss during the test is denoted by ∆*m*. The test results in an open circuit (Test 1) were also listed for comparison.

In Group ii, *T_SV_* and *T_TR_* showed an increasing trend with the increase of discharge current. Moreover, *T_max_* decreased with the increase of discharge current, which might be due to the thermal runaway behaviors shown in Figure 3. When discharged with 3 A, 4 A, and 6 A, the cell temperature was too low to ignite the solid phase particles and combustion aerosol. Therefore, there were no sparks or flames observed in those tests. In the meantime, the cell with a higher discharge current showed less mass loss and lower maximum temperature rise. Compared to the battery without discharge, the thermal runaway of the cells in the discharge condition was not very violent and had lower temperature.

When discharged at a high rate, more capacity was consumed in comparison with the samples discharged at a low rate. Thus, *C_TR_* decreased with the increasing discharge current in Group ii. In general, battery capacity has a significant effect on the thermal runaway behavior of the battery, which has been suggested by many researchers [33,34,35]. As SOC increases, more lithium metal is available in the anode to react with electrolytes to generate more flammable gases. The cell with a lower SOC has better thermal stability and thermal tolerance, as evidenced by the lower trigger temperature, lower temperature rise rates, and lower mass loss. Specifically, safety venting and thermal runaway differ in their sensitivity to the battery capacity. Safety venting was mainly triggered by the reaction between the intercalated lithium and electrolyte solvents. Compared to *T_TR_*, SOC shows an inconspicuous effect on the *T_SV_* [38]. Therefore, *T_SV_* barely changed when the cell discharged at a low rate (1 A and 2 A). In this regard, the key parameters in Group ii are significantly affected by capacity change, and the effect of the discharge current is unclear.

**Figure 5:**
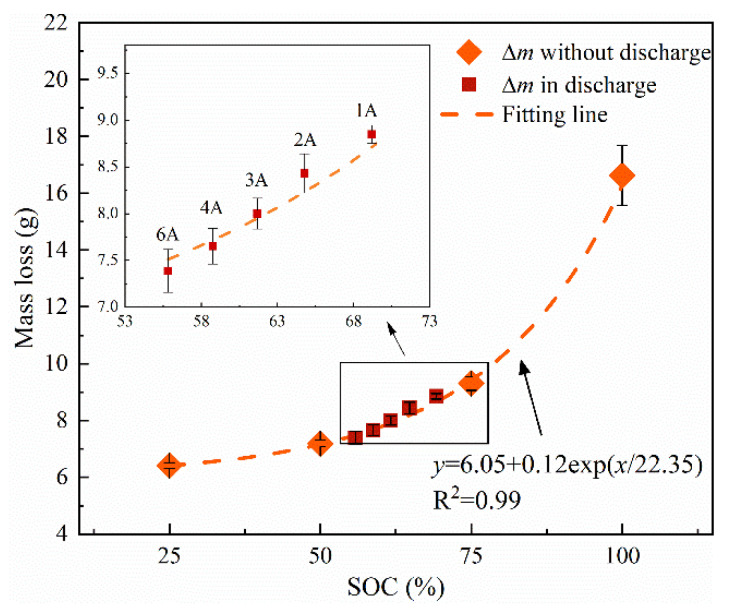
Mass loss during thermal runaway process as a function of SOC.

The total mass losses during the thermal runaway process are listed in Table 4. To identify the effect of the capacity, the batteries with various SOCs (25%, 50%, 75%, and 100%) were tested in the same condition in an open circuit. A relationship between ∆*m* and SOC from those tests is found to be ∆*m* = 6.05 + 0.12exp(*SOC*/22.35), which can be seen in Figure 5. The ∆*m* values in different discharge currents are also depicted in Figure 5, while those corresponding *C_TR_* values are presented as percentage of nominal capacity (SOC). This exponential relationship between ∆*m* and SOC in an open circuit can fit the discharge data with satisfying accuracy. The result indicates that the discharge currents can hardly deviate from the mass loss during the thermal runaway, while the capacity inside the battery plays a dominant role.

The onset times of safety venting and thermal runaway are presented in Table 4. Compared to in an open circuit, *t_SV_* and *t_TR_* were both decreased in the discharge condition, which means the batteries are more vulnerable to thermal runaway in the operational state. Moreover, the trigger temperature of safety venting and thermal runaway presents an opposite trend with the onset time in Group ii. In other words, the cells in the operational state reached higher temperatures in a shorter heating time, which indicates that the discharge current brings extra energy and accelerated the failure of the LIBs.

**Figure 6:**
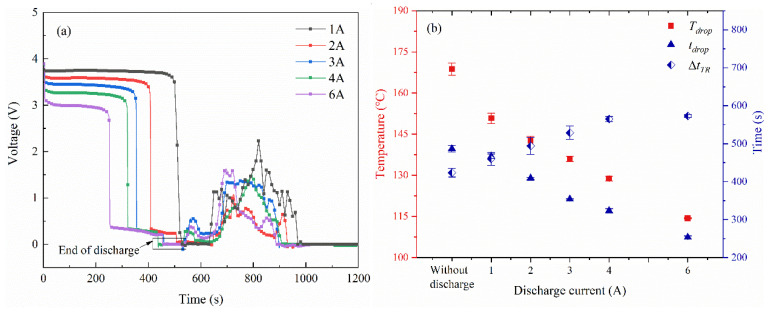
The voltage characteristics of the battery across thermal runaway when discharged with same *C*_0_ (Group ii). (**a**) The voltage variation. (**b**) Average values of *T_drop_*, *t_drop_*, and ∆*t_TR_*.

The voltage characteristics of the battery during thermal runaway in Group ii are shown in Figure 6. Before the test, all the samples kept an open-circuit voltage *U_OCV_* of 3.9 V. Later, a slight voltage decline was observed at the beginning of the test. When the current *I* flows through the battery, the operating voltage *U* deviates from *U_OCV_* due to electrochemical polarization energy. The voltage difference ∆*U = U_OCV_* − *U* = *IR*, where *R* represents the resistance caused by nickel ribbon, contact resistance, and the internal resistance of the battery. Therefore, the slight voltage decline is proportional to the discharge current. After that, a steep voltage drop is observed with the increasing temperature. It can be seen the voltage drops earlier as the discharge current increases. To investigate the voltage variation during the thermal runaway, the average values of key parameters are present in Figure 6b. The corresponding temperature for the voltage drop is defined as *T_drop_*, where *t_drop_* represents the time interval from when the LIB starts to be heated to the voltage drop, and ∆*t_TR_* is defined to describe the time interval that each experiment takes from the voltage drop to the onset of thermal runaway. As the increase of discharge current, *T_drop_* showed a significant decrease. It can be concluded that the self-heating reaction inside the battery was accelerated under discharge conditions. Moreover, *t_drop_* was severely reduced with the increasing current. Compared to the cell in an open circuit, the *t_drop_* value of the cell in 6 A discharge was almost half reduced. Besides, a larger ∆*t_TR_* was observed at the battery with a higher discharge rate. After the voltage drop, it takes about 420–520 s for the onset of thermal runaway. Therefore, the voltage drop can be used as an early warning signal to predict the occurrence of the coming thermal runaway [48].

### 3.3. Discharge Tests on the LIBs with the Same C_TR_

The capacity variations during the thermal runaway process in Group iii are shown in Table 5. In this way, the average *C_TR_* is 1655.67 ± 6.5 mAh, and the capacity difference is within ±1%. Therefore, *C_TR_* could be assumed to be roughly 1650 mAh, and the effect of capacity can be ignored in Group iii.

**Figure 7:**
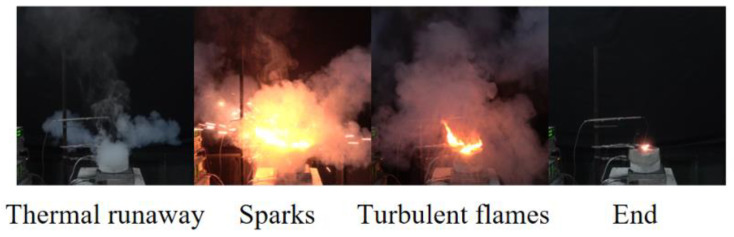
Typical thermal runaway behaviors under different discharge conditions in Group iii.

The failure processes of the cells under different discharge currents behaved similarly, and representative characteristic diagrams are shown in Figure 7. Considerable combustion gases were ejected from the safety vent ports accompanied by sparks, which then formed into a leaping flame.

**Figure 8:**
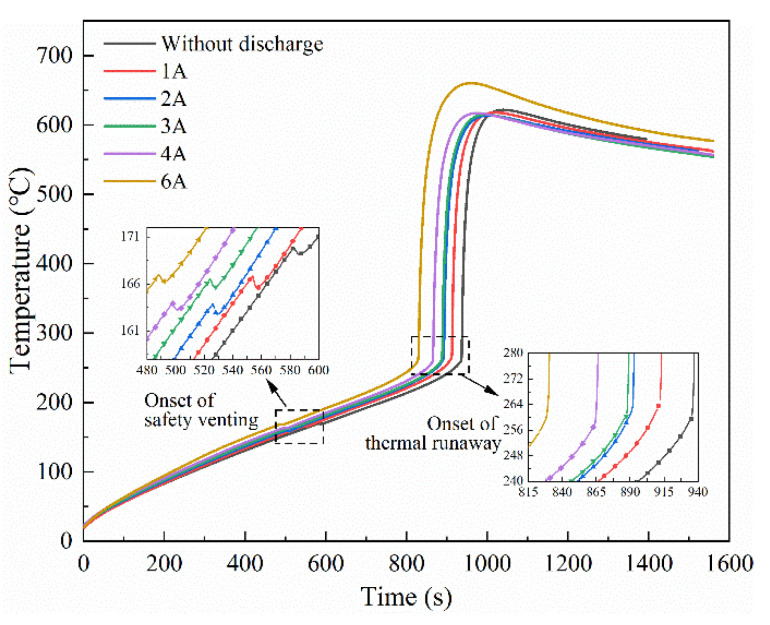
Typical temperature variation of the battery at different discharge currents in Group iii.

Typical temperature variations of the battery in Group iii are shown in Figure 8, the test results in an open circuit (Test 1) were also included for comparison. It can be seen that only a slight distinction is found in the temperature profile of the battery. The key parameters of the thermal runaway process are listed in Table 6. It can be seen that the *T_SV_*, *T_TR_*, *T_max_*, (d*T*/d*t*)*_max,_* and ∆*m* values were hardly changed with the increase of discharge current. The result indicates that the internal exothermic reactions during thermal runaway were determined by the capacity inside the battery, and the discharge current does not change it.

**Figure 9:**
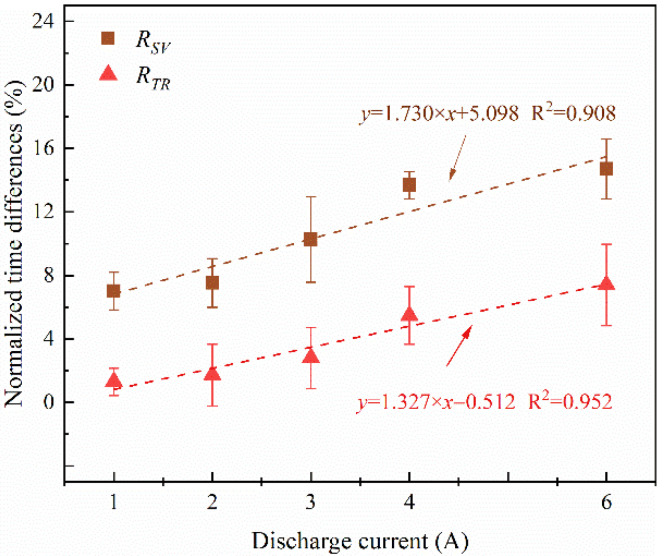
Normalized time reductions in safety venting and thermal runaway under different discharge currents in Group iii.

The onset times of safety venting and thermal runaway are presented in Table 6. *t_SV_* and *t_TR_* were both reduced with the increase of discharge current, which indicated that the batteries experienced earlier safety venting and thermal runaway in discharge condition. To explore the effect of the discharge current, the time reductions of safety venting and thermal runaway were analyzed. The onset time of safety venting and thermal runaway are highly dependent on the heating condition of the sample, and the significance of the time reductions cannot be appreciated by direct comparison. Consequently, the time reductions to safety venting and thermal runaway of the battery under different discharge currents were normalized as follows:

(1) $$R_{SV}^{i}=\frac{t_{SV}^{0}-t_{SV}^{i}}{t_{SV}^{0}}\times 100\%,\;\;i=1,2,3,4,6$$

(2) $$R_{TR}^{i}=\frac{t_{TR}^{0}-t_{TR}^{i}}{t_{TR}^{0}}\times 100\%,\;\;i=1,2,3,4,6$$

where *R* is the normalized time reduction, *t*^0^ means the onset time of the cell in an open circuit, superscript *i* means the value of discharge current, subscripts *SV* represent safety venting, and subscripts *TR* represent thermal runaway. The calculation results are depicted in Figure 9. It can be seen that *R_SV_* is always higher than *R_TR_* with different discharge currents, which indicates that the discharge current has a relatively large effect on the onset of safety venting. The results show that *R_SV_* and *R_TR_* are both proportional to the value of discharge currents, and their relationships are shown in Figure 9. Compared to the cell in an open circuit, *t_SV_* decreased around 7.01–14.70% while *t_TR_* decreased around 1.27–7.4% when discharged 1 to 6 A. Consequently, the maximum discharge current must be restricted strictly to avoid the deterioration of battery safety threatening. Different strategies should be adopted by battery management system (BMS) based on the discharge rate and the operation time to realize precise monitoring.

**Figure 10:**
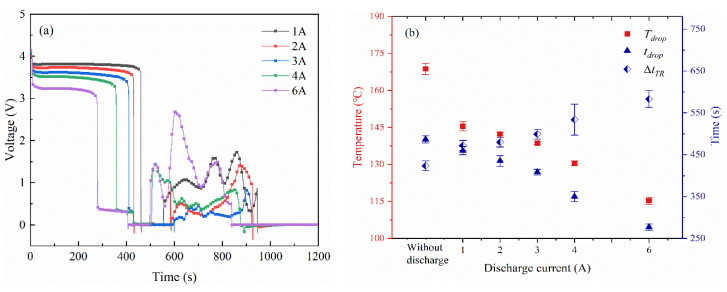
The voltage characteristics of the battery across thermal runaway when discharged with same *C_TR_* (Group iii). (**a**) The voltage variation. (**b**) Average values of *T_drop_*, *t_drop_*, and ∆*t_TR_*.

The voltage characteristics of the battery during thermal runaway in Group iii are shown in Figure 10. Compared to the electrically isolated LIBs, the battery in the discharge condition experiences an earlier voltage drop. It can be concluded that the fire risk of the battery was elevated in the operational state. More strict strategies, e.g., measures to enhance heat dissipation and sensitive temperature detection, should be taken for battery safety use under high-rate discharge.

## 4. Discussion

To quantify the thermal difference between batteries in the open-circuit and the operational state, the heat generation of LIBs in discharge tests (i.e., Group iii) was analyzed to offer advice for battery safety management modification.

For the cell in discharge condition, the energy brought by external electric power (*P_ex_*) and discharge current (*P_I_*), as well as the heat generated inside the battery due to high temperature (*P_IHG_*) were used to enhance the temperature of the battery and hollow copper cylinder. Partial energy was dissipated to the environment (*P_loss_*). The *Bi* number estimated in this study by approximating the sample as an aluminum cylinder is about 0.002. When *Bi* < 0.1, the thermal resistance and the temperature gradient of cells are negligible. Therefore, the temperature inside the battery was considered to be spatially uniform. The energy conservation equation is as follows:

(3) $$P_{ex}+P_{IHG}+P_{I}=c_{Cu}m_{Cu}\frac{dT_{Cu}}{dt}+c_{LIB}m_{LIB}\frac{dT}{dt}+P_{loss}$$

**Figure 11:**
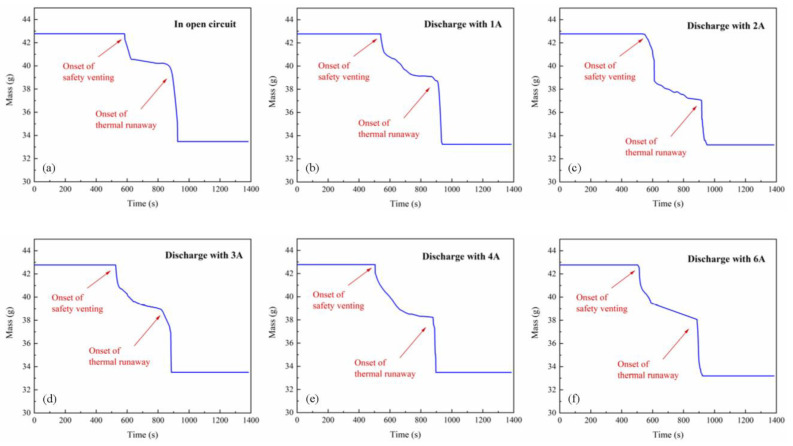
Representative LIB mass histories used in the calculation of discharge energetics. (**a**) The LIB in open circuit; (**b**) The LIB discharge with 1 A; (**c**) The LIB discharge with 2 A; (**d**) The LIB discharge with 3 A; (**e**) The LIB discharge with 4 A; (**f**) The LIB discharge with 1 A.

*m_Cu_* represents the mass of the copper cylinder, which was a constant. The heat capacity of the copper slug can be defined as *c_Cu_* =2.79 × 10^−1^ + 4.42 × 10^−1^*T* − 4.92 × 10^−7^*T*^2^ + 2.20 × 10^−10^*T*^3^ + 1.08 × 10^−3^*T*^−2^ J/(g·K) [49]. *T_Cu_* is the temperature of the hollow copper, which is equal to the battery temperature (*T*) since there is good thermal contact between the copper and the LIB. *m_LIB_* represents the mass of the battery. The *m_LIB_* histories used in the calculation are shown in Figure 11. The rebounds of those curves were removed since they cannot represent the real mass. Here, *c_LIB_* is defined as the heat capacity of the battery, which is determined to 1.1 J/(g·K) by the ARC.

*P_ex_* was kept at a constant value of 20 W during the test. *P_loss_* represents the rate of thermal dissipation from the cell and the copper cylinder to the insulation and ambient air. To determine the relationship between *P_loss_* and the cell temperature, a set of experiments was carried out where an LIB sample was replaced with a failed cell. During these tests, *P_IHG_* and *P_I_* were kept to zero, the mass of the failed cell hardly changed, and the heat capacity of the failed cell was considered to be the same as that of the normal cell. Therefore, the value of *P_loss_* can be computed from the measured data. When the cell was heated by 20W, *P_loss_* can be obtained as *P_loss_* = −5.88 + 7.3 × 10^−2^*T* − 7.5 × 10^−5^*T*^2^ + 7.07 × 10^−8^*T*^3^. Details of the fitting procedure can be found in our earlier publication [50].

**Figure 12:**
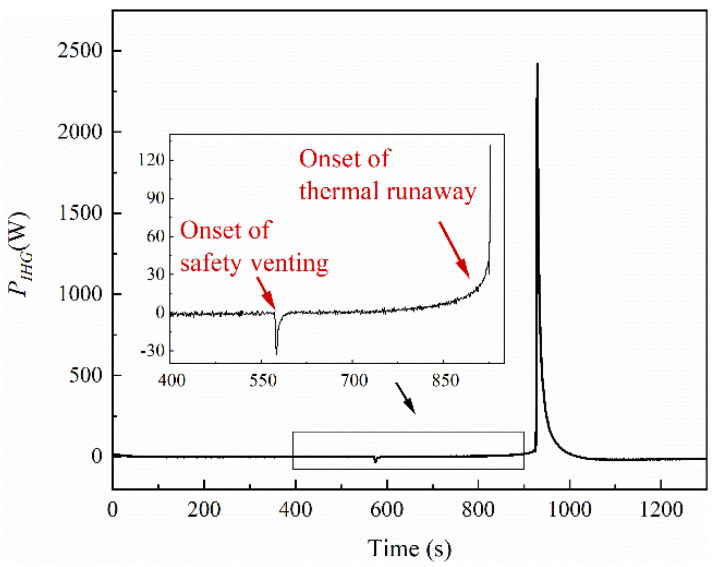
Heat generation by the processes inside LIBs during the thermal runaway process in an open circuit.

*P_IHG_* is the power of heat generation inside the battery during its thermal runaway. For the cell in an open circuit, *P_I_* was kept at zero. Using the data from Test 1, *P_IHG_* can be calculated by Equation (3). *P_IHG_* is plotted as a function of time shown in Figure 12. A small endothermic peak was observed at the time of onset of safety venting, which is speculated to be associated with the vaporization of the electrolyte.

In this way, *P_I_* can be calculated once the other term in Equation (3) was obtained. Considering that the discharge current cannot impact the heat generation of the LIB during thermal runaway, the total energy produced inside the battery due to discharge current (*Q_I_*) was calculated as follows:

(4) $$Q_{I}=\int_{0}^{t_{TR}}P_{I}dt.$$

**Figure 13:**
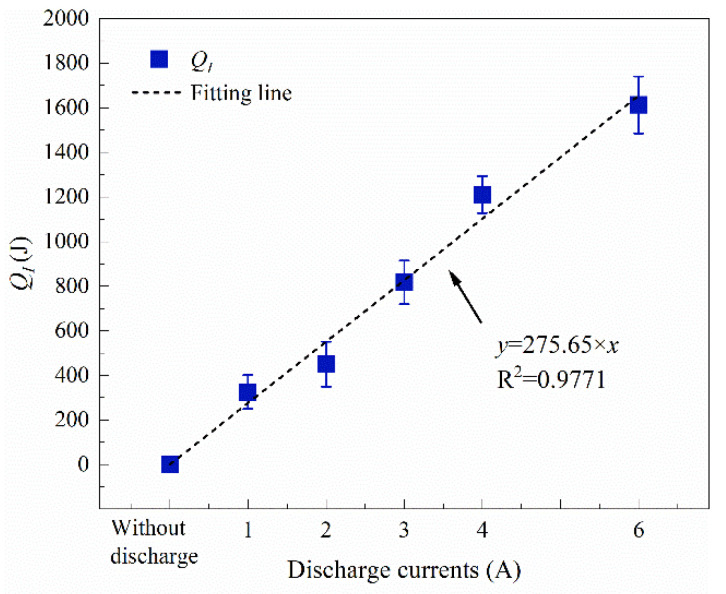
Total energy produced inside the battery vs. discharge current.

The total energy produced inside the batteries is determined to be 0.3–1.6 kJ when discharged at 1 to 6 A, which is shown in Figure 13. It can be seen that *Q_I_* increased linearly with the increasing discharge current, with a fitting correlation of *y* = 275.65 × *x*. Moreover, if the energy was applied to the cell neglecting heat loss, the temperature of the battery will rise 7 °C, 10 °C, 17 °C, 26 °C, and 34 °C, respectively, with a discharge rate of 1 A, 2 A, 3 A, 4 A, and 6 A. In practical cases, LIBs are usually assembled into large arrays or battery packs to meet high-power requirements. A great amount of heat would be generated and accumulated inside the module, greatly increasing the risks of fires and explosion accidents.

## 5. Conclusions

In this work, 18650-type LIBs with Li(Ni_0.33_Co_0.33_Mn_0.33_)O_2_ cathode were tested in a variety of discharge conditions. The battery experienced an inevitable capacity decrease during the discharge process. Therefore, comparative experiments were performed to investigate the effect of the residual capacity and discharge currents on the thermal runaway process of the battery. The main conclusions of this study can be summarized as follows:

(1) Results show that the key parameters during the failure process, such as the onset temperature of safety venting and thermal runaway, peak temperature, peak temperature rise rate, and total mass loss, are ultimately determined by the capacity inside the battery, and discharge current can hardly change it.

(2) Compared to the cell in an open circuit, the thermal runaway process of the cell was accelerated in the operational state. Moreover, the time to thermal runaway is decreased with the increase of discharge current. More strict strategies, e.g., measures to enhance heat dissipation and sensitive temperature detection, should be taken for battery safety use under high-rate discharge.

(3) Discharge currents can produce extra energy to accelerate the thermal runaway process. The total heat generations inside the batteries due to discharge current are calculated to be 0.3–1.6 kJ when discharge at 1–6 A. An empirical formula is deduced for estimating the heat production regarding the different discharge currents.

This work is significant for the safety design of the battery in engineering applications since it provides a comprehensive understanding of the thermal hazards of LIBs in the operational state. The operating current must be restricted strictly to avoid undesirable heat generations. Different strategies should be adopted by BMS based on the discharge rate and the operation time to realize precise monitoring. The conclusion from this study should be taken into consideration to help the safety application of LIB and improve the reliability of the BMS.

## Figures and Tables

**Table 1:** The list of typical LIB fire accidents in the operational state.

Date	Location	Incident
July 2021	Hangzhou, China	An electro mobile caught fire while in motion [24].
April 2019	Cordova, Spain	The battery inside an e-cigarette exploded when using [25].
June 2018	California, USA	A Tesla car released smoke while being driven [26].
January 2016	Mudeungsan, Korea	A Renault-Samsung electric vehicle caught fire while driving [27].

**Table 2:** Test conditions.

Group No	Test No	Discharge Current (A)	*C*_0_ (mAh)
i	1	—	1650
ii	2	1	1650
	3	2	
	4	3	
	5	4	
	6	6	
iii	7	1	1775
	8	2	1900
	9	3	2000
	10	4	2050
	11	6	2125

**Table 3:** Capacity variation during the thermal runaway process in Group ii.

Test No	Discharge Current (A)	*C*_0_ (mAh)	Energy Consumption ^1^ (mAh)	*C_TR_* (mAh)
2	1	1650	127.68 ± 0.92	1522.32 ± 0.92
3	2	1650	224.66 ± 2.66	1425.34 ± 2.66
4	3	1650	292.98 ± 1.98	1357.02 ± 1.98
5	4	1650	357.24 ± 5.96	1292.76 ± 5.96
6	6	1650	422.10 ± 6.65	1227.90 ± 6.65

^1^ The uncertainties reported in this manuscript were calculated from the experimental data among the repeated tests.

**Table 4:** Key parameters of the thermal runaway at different discharge currents in Group ii.

Test No	Discharge Current /A	Onset of Safety Venting		Onset of Thermal Runaway		*T_max_*/°C	(d*T*/d*t*)*_max_*/°C·s^−1^	∆*m*/g
		*t_SV_*/s	*T_SV_*/°C	*t_TR_*/s	*T_TR_*/°C			
1	—	585 ± 12	168.75 ± 2.26	932 ± 5	262.11 ± 0.35	621.61 ± 1.74	36.68 ± 1.17	9.30 ± 0.24
2	1	554 ± 8	170.55 ± 5.01	917 ± 15	272.86 ± 3.01	620.49 ± 13.69	29.67 ± 1.77	8.85 ± 0.10
3	2	546 ± 2	169.50 ± 3.81	905 ± 11	274.19 ± 4.03	618.98 ± 4.63	23.87 ± 1.92	8.43 ± 0.21
4	3	541 ± 5	174.57 ± 5.13	900 ± 12	282.77 ± 3.2	611.43 ± 8.31	19.73 ± 2.48	8.00 ± 0.17
5	4	540 ± 10	177.19 ± 2.53	897 ± 3	282.92 ± 2.25	600.87 ± 1.33	17.24 ± 4.00	7.65 ± 0.19
6	6	538 ± 7	180.50 ± 1.96	872 ± 1	286.47 ± 7.41	585.26 ± 3.86	13.68 ± 0.40	7.39 ± 0.23

**Table 5:** Capacity variation during the thermal runaway process in Group iii.

Test No	Discharge Current (A)	*C*_0_ (mAh)	Energy Consumption (mAh)	*C_TR_* (mAh)
7	1	1775	126.89 ± 2.69	1648.11 ± 2.69
8	2	1900	240.68 ± 7.01	1659.32 ± 7.01
9	3	2000	339.07 ± 5.39	1660.93 ± 5.39
10	4	2050	392.42 ± 3.56	1657.58 ± 3.56
11	6	2150	457.57 ± 4.68	1662.43 ± 4.68

**Table 6:** Key parameters of the thermal runaway at different discharge currents in Group iii.

Test No	Discharge Current /A	Onset of Safety Venting		Onset of Thermal Runaway		*T_max_*/°C	(d*T*/d*t*)*_max_*/°C·s^−1^	∆*m*/g
		*t_SV_*/s	*T_SV_*/°C	*t_TR_*/s	*T_TR_*/°C			
1	—	585 ± 12	168.75 ± 2.26	932 ± 5	262.11 ± 0.35	621.61 ± 1.74	36.68 ± 1.17	9.30 ± 0.24
7	1	542 ± 7	165.28 ± 6.34	920 ± 8	264.01 ± 1.69	621.16 ± 2.90	36.73 ± 2.28	9.52 ± 0.09
8	2	541 ± 9	165.28 ± 6.34	916 ± 18	264.11 ± 1.57	618.37 ± 4.73	35.27 ± 0.30	9.57 ± 0.29
9	3	525 ± 16	166.89 ± 2.45	906 ± 18	264.30 ± 1.85	628.79 ± 14.18	33.70 ± 0.49	9.26 ± 0.15
10	4	505 ± 5	164.85 ± 3.34	881 ± 17	264.68 ± 2.05	619.14 ± 2.36	36.18 ± 2.64	9.30 ± 0.06
11	6	499 ± 11	166.97 ± 1.65	863 ± 24	263.09 ± 2.66	627.94 ± 18.78	33.98 ± 1.72	9.58 ± 0.30

## Data Availability

The data presented in this study are available on request from the corresponding author. The data are not publicly available due to possible proprietary concerns related to patent protection on this work.
